# Data from a national survey of United States primary care physicians on genetic risk scores for common disease prevention

**DOI:** 10.1016/j.dib.2023.109930

**Published:** 2023-12-08

**Authors:** Charles A. Brunette, Elizabeth J. Harris, Ashley A. Antwi, Amy A. Lemke, Benjamin J. Kerman, Jason L. Vassy

**Affiliations:** a Veterans Affairs Boston Healthcare System, Boston, MA, USA; b Department of Medicine, Harvard Medical School, Boston, MA, USA; c Norton Children's Research Institute, University of Louisville School of Medicine, Louisville, KY, USA; d Division of General Internal Medicine and Primary Care, Brigham and Women's Hospital, Boston, MA, USA; e Precision Population Health, Ariadne Labs, Boston, MA, USA

**Keywords:** Cardiovascular disease, Disease prevention, Health disparities, Polygenic risk scores, Prostate cancer

## Abstract

Genetic risk scores (GRS) are an emerging and rapidly evolving genomic medicine innovation that may contribute to more precise risk stratification for disease prevention. Inclusion of GRS in routine medical care is imminent, and understanding how physicians perceive and intend to utilize GRS in practice is an important first step in facilitating uptake. This dataset was derived from an electronic survey and comprises one of the first, largest, and broadest samples of United States primary care physician perceptions on the clinical decision-making, benefits, barriers, and utility of GRS to date. The dataset is nearly complete (<1% missing data) and contains responses from 369 PCPs spanning 58 column variables. The public repository includes minimally filtered, de-identified data, all underlying survey versions and items, a data dictionary, and associated analytic files.

Specifications Table

**Table:** 

Subject	Health and medical science: Clinical genetics; Cardiology and cardiovascular medicine; Oncology
Specific subject area	Personalized medicine, precision medicine, genetic risk scores (GRS) in primary care, physician decision-making.
Data format	Filtered (de-identified) raw dataset (CSV); De-identification approach and data dictionary (Excel); Primary analytic files for regression analysis and latent class variable modeling (R); English-language version of questionnaire (.pdf).
Type of data	Tabular data; Data dictionary; Primary analytic files; Questionnaire/Survey
Data collection	Data were acquired between April 18, 2021 and August 27, 2021 from a random sample of United States primary care physicians from the IQVIA ONEKEY database via email invitation and an online survey instrument (Qualtrics, Provo, UT). Randomized clinical scenarios were developed specifically for this questionnaire. Benefits, barriers, and utility items were adapted from the Physician Survey on Cancer Susceptibility Testing, Survey of Primary Care Physicians’ Recommendations & Practice for Cancer Screening, and work by Grant et al., 2009, Mikat-Stevens et al., 2015, Christensen et al., 2016, and Lemke et al., 2020. The survey protocol was approved by the Harvard Longwood Campus IRB (#IRB20–2098) and all participants provided informed consent.
Data source location	Data collected from primary care physicians across the United States, by geographic region (Midwest, Northeast, South, West).
Data accessibility	Repository name: Harvard Dataverse Dataset: Data from National PCP Survey on GRS https://doi.org/10.7910/DVN/QFITQF Direct URL to data: https://doi.org/10.7910/DVN/QFITQF
Related research article(s)	B.J. Kerman, C.A. Brunette, E.J. Harris, A.A. Antwi, A.A. Lemke, J.L. Vassy, Primary care physician use of patient race and polygenic risk scores in medical decision-making, Genet. Med. 25 (2023). https://doi.org/10.1016/j.gim.2023.100800 J.L. Vassy, B.J. Kerman, E.J. Harris, A.A. Lemke, M.L. Clayman, A.A. Antwi, K. MacIsaac, T. Yi, C.A. Brunette, Perceived benefits and barriers to implementing precision preventive care: Results of a national physician survey, Eur. J. Hum. Genet. (2023) 1–8. https://doi.org/10.1038/s41431-023-01318-8

## 1 Value of the Data

• Genetic risk scores (GRS, also known as polygenic risk scores, PRS) are an emerging and rapidly evolving genomic medicine innovation that may contribute to more precise risk stratification for disease prevention. Specifically, a GRS is a measure of an individual's inherited risk of a certain health-related condition based on the aggregated effects of hundreds to millions of genetic variants across the human genome.
• Inclusion of GRS in routine medical care remains largely investigational but is likely imminent for many diseases as models continually improve, scores are integrated with other efficacious risk stratification tools, and costs for genetic testing decline. Therefore, understanding how physicians perceive and might intend to utilize GRS in practice is an important first step in facilitating informed uptake and implementation.
• This dataset comprises one of the first, largest, and broadest samples of United States primary care physician perceptions on the clinical decision-making, benefits, barriers, and utility of GRS within the landscape of preventive medicine to date.
• Public availability of this dataset, survey, and associated analytic files will allow other interested researchers in the field of precision medicine and implementation science to: 1) more easily review and replicate our findings, 2) perform additional analyses using different research perspectives, questions, and/or analytic methods, 3) allow ease of access to raw data in the event our outcomes are included in any future meta-analytic assessments, and 4) utilize the data as a comparator and/or validation set for the administration of our questionnaire items to other samples.

## 2 Data Description

### 2.1 Background

The data presented here is associated with the protocol *A National Survey of United States Primary Care Physicians on Genetic Risk Scores for Common Disease Prevention* (Harvard Longwood Campus Institutional Review Board approved Protocol #20-2098). The protocol outlines a study that was developed to: 1) better understand whether primary care physicians (PCPs) would incorporate genetic risk scores (GRS) into their medical decision-making across a series of clinical vignettes, 2) determine whether their use of GRS in these vignettes would vary based on the reported race of the hypothetical patient in each scenario, and 3) ascertain general perceptions of the benefits, barriers, and clinical usefulness of GRS. Background, analysis, interpretation, and discussion of the randomized experiment associated with this protocol, involving GRS, clinical decision-making, and patient race, is described in Kerman et al. [1] whereas general perceptions on the benefits, barriers, and utility of GRS is reported in Vassy et al. [2].

Public data [3] from this protocol includes filtered, de-identified survey data from a United States sample of 369 PCPs spanning 58 column variables (*data_clean.csv*). Data comprises 1) basic survey response metrics, 2) demographic information, 3) self-reported genetics training, 4) clinical decision-making scenarios, and 5) items pertaining to perceived benefits, barriers, disease applicability, and utility of GRS. The dataset is nearly complete, containing few data points coded as *‘missing’* (∼0.24% of total dataset). A description of the de-identification process as well as a data dictionary detailing each column variable, its related survey item, and item response options accompanies the raw dataset (*data_de-identifcation_steps_and_data_dictionary.xlsx*). Respondents were randomly assigned to receive one of 4 versions of the survey. Complete copies of all survey versions (*Versions 1–4* include identical questions, except for variation of patient reported race among the clinical scenarios), are available in the public repository (*qualtrics_survey_v1-4.pdf*). To allow for review and replicability of our main findings, two analytic files from the above outcomes manuscripts are also included (*gim_gee_modeling_primary.r; ejhg_lca_modeling_primary.r*).

### 2.2 Participant demographic information

**Fig. 1:**
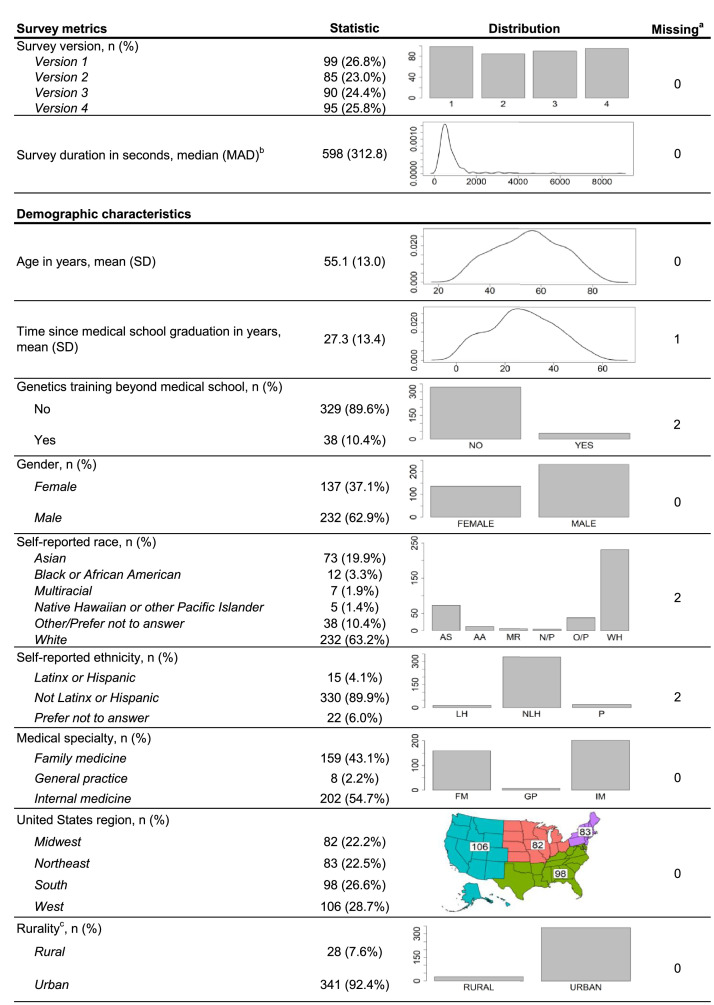
Survey metrics and demographic characteristics of the data. ^a^Total number of missing data points in the variable column. Missing data coded as ‘*missing*’. ^b^Survey duration descriptive statistics presented as median and median absolute deviation (MAD) due to heavy positive skew. Distribution figure truncated at 10,000 s for visibility, total n represented = 359. ^c^Rurality inferred from practice 5-digit ZIP code and USDA Rural-Urban Commuting Area Codes categorizations (4–10 considered rural; Micropolitan area with primary flow to urban clusters < 50,000 population). ZIP code not provided as part of this dataset due to de-identification requirements.

Fig. 1 shows the survey metric and demographic characteristics from the raw data file. Survey versions were distributed uniformly across the sample, with *Version 1* being completed by the most participants (99/369, 26.8%) and *Version 2* being completed by the fewest (85/369, 23.0%). Large variation in survey duration (the amount of time between beginning the survey and completing it) was observed across the sample, spanning completion in less than two minutes (105 s) to over a full week (nearly 9 days). Age and time since medical school graduation were relatively normally distributed across the sample and ranged from 28 to 83 years old and 1–59 years, respectively. Only 10.4% (38/367) of participants reported engagement in any genetics-focused training beyond medical school. Fewer respondents self-identified as women (137/369, 37.1%), self-reported their race as something other than White (135/367, 36.8%), or self-reported their ethnicity as Latinx or Hispanic (15/367, 4.1%). Most participants identified their medical specialty as internal medicine (202/369, 54.7%), compared to family medicine (159/369, 43.1%) and general practice (8/369, 2.2%). Respondents were well-dispersed across United States Regions [4], with greatest representation from the western United States (106/369, 28.7%). A majority of participants were from urban (341/369, 92.4%) settings as derived from medical practice 5-digit ZIP codes and United States Department of Agriculture (USDA) Rural-Urban Commuting Area Code categorizations [5]. Race, ethnicity, and genetics training beyond medical school were obtained directly from survey respondents. All other demographic variables were acquired from the IQVIA *ONEKEY* database (described in the methods below).

### 2.3 Clinical vignettes

**Fig. 2:**
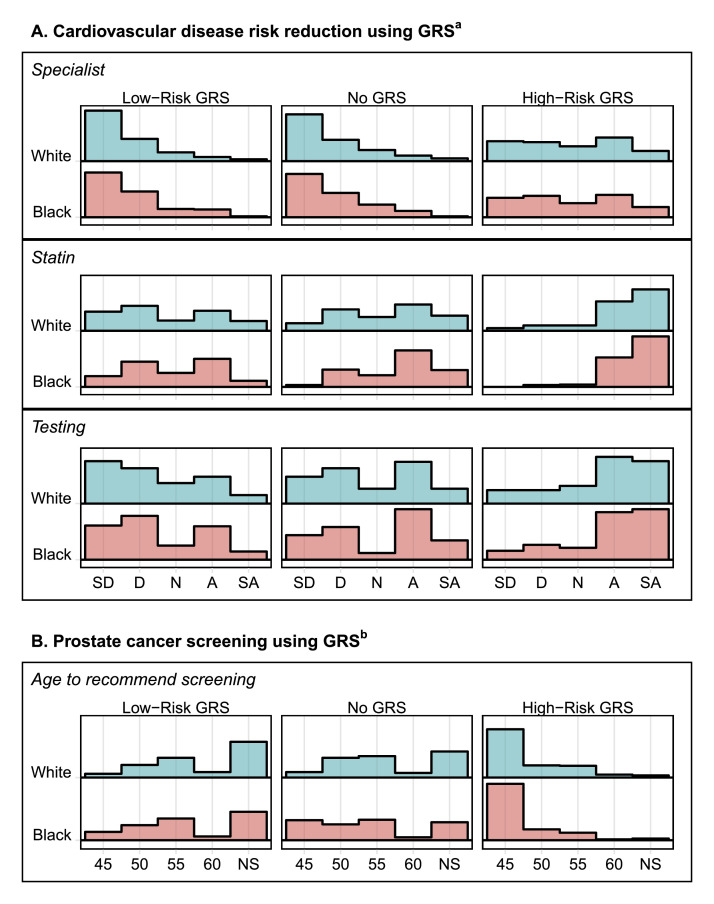
Distributions of participant response data for cardiovascular disease prevention and prostate cancer screening clinical scenarios stratified by randomized hypothetical patient reported race (Black or White) and genetic risk score (GRS) information (low-risk GRS, no GRS, and high-risk GRS). ^a^Responses to cardiovascular disease scenarios to 1) recommend referral to a specialist, 2) recommend a statin prescription, and 3) recommend additional cardiac testing; Total participants completing items for Black patient scenario, *N* = 180; Total participants completing items for White patient scenario, *N* = 189; *SD = Strongly disagree, D = Disagree, N = Neither agree nor disagree, A = Agree, SA = Strongly agree*. ^b^Responses to prostate cancer screening scenarios and age to recommend prostate cancer screening; Total participants completing items for Black patient scenario, *N* = 185; Total participants completing items for White patient scenario, *N* = 184; *45 = recommend screening at age 45, 50 = recommend screening at age 50, 55 = recommend screening at age 55, 60 = recommend screening at age 60*, NS = *do not recommend screening at any age*.

Fig. 2 displays response distributions associated with the randomized clinical vignettes stratified by disease scenario and decision-making items, race reported for the hypothetical patient in the vignette (Black or White), and GRS risk status. A total of 369 participants completed the clinical vignette survey items. Cardiovascular disease prevention items were administered with Likert scale options ranging from *Strongly disagree (1)* to *Strongly agree (5)* for whether to recommend specialist referral, statin prescription, and additional cardiac testing. A total of 180 participants completed scenario items including a Black patient (survey *Versions 2 and 4*) and 189 participants completed scenario items including a White patient (survey *Versions 1 and 3*) within each cardiovascular disease prevention vignette. In general, fewer participants agreed (*Agreed or Strongly agreed*) that referral to a specialist (31/369, 8.4%), a statin prescription (140/369, 37.9%), and additional cardiac testing (106/369, 28.7%) was necessary for a patient with a low-risk GRS and more participants agreed that referral to a specialist (140/369, 37.9%), a statin prescription (330/369, 89.4%), and additional cardiac testing (258/369, 70.0%) was necessary for a patient with a high-risk GRS, versus patient scenarios where no GRS information was provided (33/369, 8.9%; 207/369, 56.1%; 174/369, 47.2%). Across all GRS risk scenarios, a greater proportion of responses (each participant contributed a response to each clinical scenario for low-risk GRS, no GRS, and high-risk GRS, respectively) agreed that referral to a specialist (100/540, 18.5%), a statin prescription (358/540, 66.3%), and additional cardiac testing (282/540, 52.2%) was necessary for a Black patient versus a White patient (104/567, 18.3%; 319/567, 56.2%; 256/567, 45.1%). Prostate cancer screening items were administered with a Likert-type response option ranging from recommending prostate cancer screening beginning at *age 45 (1)* (subsequent options offered in five-year increments up to age 60) to *not at all (5)*. A total of 185 participants completed scenario items including a Black patient (survey *Versions 3 and 4*) and 184 participants completed scenario items including a White patient (survey *Versions 1 and 2*) within each prostate cancer screening vignette. In general, fewer respondents recommended initiating screening at any age for low-risk GRS (216/369, 58.5%) and more respondents recommended initiating screening at any age for high-risk GRS (360/369, 97.6%), compared to scenarios where no GRS information was provided (264/369, 71.5%). Across all GRS risk scenarios, a greater proportion of responses (each participant contributed a response to each clinical scenario for low-risk GRS, no GRS, and high-risk GRS, respectively) recommended initiating screening at any age for a Black patient (440/555, 79.3%) versus a White a patient (400/552, 72.5%).

### 2.4 General perceptions

**Fig. 3:**
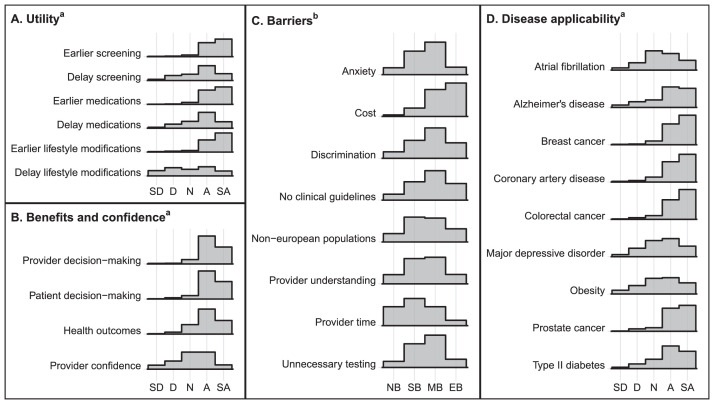
Distributions of participant response data for perceived utility, benefits and confidence, barriers, and disease applicability for genetic risk scores. ^a^Response options and abbreviations for utility, benefits and confidence, and disease applicability: *SD = Strongly disagree, D = Disagree, N = Neither agree nor disagree, A = Agree, SA = Strongly agree*. ^b^Response options and abbreviations for barriers: *NB = Not a barrier, SB = Somewhat of a barrier, MB = Moderate barrier, EB = Extreme barrier*.

Fig. 3 presents response distributions related to the clinical usefulness, benefits, barriers, and disease applicability items, which were identical across survey versions. Utility, benefits, and disease applicability items were administered with Likert scale options ranging from *Strongly disagree (1)* to *Strongly agree (5)*. Respondents with available data agreed (*Agreed or Strongly agreed*) that GRS would be more useful in initiating earlier interventions, including screening (341/369, 92.4%), preventive medications (342/369, 92.7%), and lifestyle modifications (337/369, 91.3%) versus delaying them (234/369, 63.4%; 241/369, 65.3%; 152/369, 41.2%). Most respondents agreed that GRS could enhance provider decision-making (326/367, 88.8%), patient decision-making (331/367, 90.2%), and patient health outcomes (281/367, 76.6%), but fewer than half of PCPs agreed they would feel confident (155/367, 42.2%) using GRS in clinical practice. Participants expressed the most interest in using GRS for breast (334/367, 91.0%), colorectal (328/367, 89.4%), and prostate (325/367, 88.6%) cancer and were least interested in using GRS for major depressive disorder (190/367, 51.8%), obesity (182/367, 49.6%), and atrial fibrillation (175/367, 47.7%) of the nine diseases included in the survey. Barrier items were administered with Likert-type response options ranging from *Not a barrier (1)* to *Extreme barrier (4)*. Of eight potential barriers named in the survey, respondents most often considered provider time to explain GRS to patients (99/368, 26.9%) as not a barrier and cost of genetic testing (175/368, 47.6%) as an extreme barrier.

## 3 Experimental Design, Materials, and Methods

**Fig. 4:**
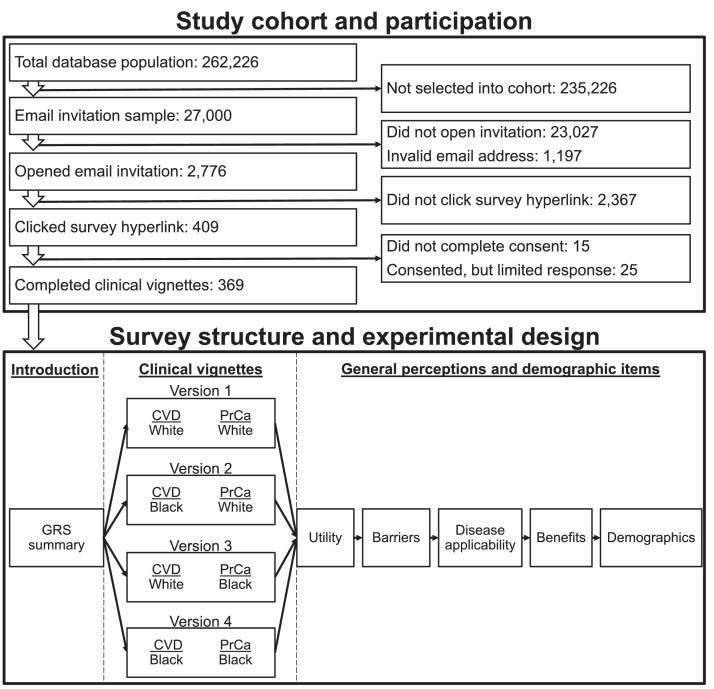
Study cohort and structure of the electronic survey. The study cohort was derived from the IQVIA *ONEKEY* database. A random sample of 27,000 individuals were selected by IQVIA to receive an electronic survey invitation by email. 369 participants completed the clinical vignettes and utility items, 368 participants completed through the barrier items, 367 participants completed through the demographic items, and 366 participants completed the survey in its entirety. 25 participants consented to participation, but did not complete the clinical vignettes entirely nor completed any other section of the survey. Hypothetical patient reported race (Black or White) was varied across clinical vignettes for each disease scenario (cardiovascular disease and prostate cancer, survey *Versions 1–4*). The electronic survey email, introduction, general perception items, and demographic questions were identical across all survey versions. Abbreviations: CVD, cardiovascular disease; GRS, genetic risk score; PrCa, prostate cancer.

To achieve protocol aims, an electronic survey was developed with an embedded randomized experiment, including identical clinical scenarios, general perception items, and demographic questions across multiple survey versions. The survey versions varied only by the reported race of the hypothetical patient described in each vignette (Black patient or White patient in either or both of the cardiovascular disease and prostate cancer survey sections). Fig. 4 details the study cohort and outlines the experimental design and structure of the survey.

### 3.1 Survey development and structure

The survey introduction included a brief summary of GRS, providing a definition, the current science, potential limitations, and a visual example. The clinical vignettes were modeled from existing PCP assessments considering risk and decision-making [6,7], and incorporated both disease-specific risk factors and genetic risk information. Presentation of the vignettes to survey respondents followed a randomized design which varied the reported race of the hypothetical patient described in each vignette (randomization methods described below). The first vignette related to cardiovascular disease risk reduction began with a brief overview of current guidelines [8] and segued into three scenarios describing a patient's cardiovascular disease risk profile (standard clinical profile and no GRS information, standard clinical profile and high-risk GRS information, and standard clinical profile and low-risk GRS information in order of presentation). After each scenario, participants were asked whether they would agree (5-point Likert scale ranging from *Strongly disagree* to *Strongly agree*) with recommending a statin prescription, ordering additional testing, and referring to a specialist. The second vignette section associated with prostate cancer screening also provided a brief overview of current clinical guidelines [9] and proceeded into three scenarios describing a patient's prostate cancer risk profile following the pattern of omitting GRS information, providing high-risk GRS information, and providing low-risk GRS information in combination with standard clinical risk factors. After each scenario, respondents were asked at what age (*45, 50, 55, 60*) they would initiate prostate cancer screening, if at all (*do not recommend screening*). General items were derived from existing instruments and assessed PCP perceptions of GRS utility (use GRS to recommend earlier or to delay disease screening, preventive medications, and lifestyle modifications; assessed on a 5-point Likert scale ranging from *Strongly disagree* to *Strongly agree*) [10], [11], [12], barriers (roadblocks to using GRS in a clinical setting; assessed on a 4-point Likert-type scale ranging from *Not a barrier* to *Extreme barrier*) [13,14], disease applicability (interest in using GRS for nine diseases in which GRS have been developed; assessed on a 5-point Likert scale ranging from *Strongly disagree* to *Strongly agree*), and benefits (use GRS to enhance patient and provider decision-making, health outcomes, and confidence in ability to use GRS; assessed on a 5-point Likert scale ranging from *Strongly disagree* to *Strongly agree*) [14]. The survey closed with items that asked respondents whether they completed any genetics-focused training beyond medical school (both formal and informal education and training) [15] and self-reported race and ethnicity. Prior to wide dissemination, the survey was piloted among a sample of eight providers and iterated on by the research team for content and clarity. The final electronic survey instrument was developed in and administered using Qualtrics software (Provo, UT).

### 3.2 Survey administration

To obtain a broad sample of PCPs, our research team worked with IQVIA to recruit potential respondents from the ONEKEY national physician database. The database includes more than 250,000 active participants who have consented to receive email invitations for a variety of purposes. The data is linked to the American Medical Association (AMA) Physician Masterfile and includes demographic information such as enrollee age, gender, geographic location, and medical specialty, among other features. Recruitment was initiated on April 18, 2021 via the dissemination of an email invitation and unique Qualtrics survey web link from IQVIA to a random sample of 27,000 ONEKEY database participants. The email invitation included a brief survey introduction, a rough estimate of required effort (∼8–10 min), an incentive offer of a $25 Amazon gift card, as well as an option to opt-out of future correspondence. Subsequent outreach was made to participants who had not opted out nor opened the initial invitation on April 27, 2021 with similar information, but included an increased incentive offer of a $50 Amazon gift card. The survey remained open until August 27, 2021. In total, 23,027 email recipients opted not to open the email invitation and 1197 invitations were sent to invalid or non-working email addresses. Of the 2776 invitees who opened the invitation, 409 clicked the survey hyperlink, and 369 ultimately consented to participation and completed, at minimum, the clinical vignette items. Invitees were randomly allocated to one of the four survey versions upon selection of the hyperlink in a 1:1:1:1 ratio using the Qualtrics software survey randomizer tool.

### 3.3 Data cleaning, merging, and derivation

All raw data was reviewed and cleaned in R (v4.0.3, R Foundation, Vienna, Austria). A total of 185 survey responses were recorded as part of the initial outreach and 256 responses were recorded as part of the second outreach attempt. Of these, 32 were duplicate responses (same unique survey link and respondent identification number) and only the first attempt was recorded as valid and retained in the overall dataset (*n* = 409). Forty additional responses did not include completed clinical vignette items (ceased at various points beginning with consent through the final scenario item) and were not retained for eventual analysis (*n* = 369). Survey version was not associated with response to the clinical vignette items (See Fig. 1 for response rate by version; total incomplete responses: *Version 1* = 5, *Version 2* = 11, *Version 3* = 11, *Version 4* = 13; X^2^ = 3.986, df = 3, *p*-value = 0.263, Cramer's *V* = 0.049).

Cleaned survey response data was subsequently merged with demographic information provided by IQVIA from the ONEKEY database using a common identifier, which included PCP age, time since medical school graduation, gender, medical specialty, and geographic data associated with each PCPs affiliated medical facility or practice city, state, and ZIP code. United States geographic region (Midwest, Northeast, South, West) was assigned according to the PCP's state of medical practice [4]. Rurality was derived from medical practice ZIP code using USDA Rural-Urban Commuting Area Codes [5] categorized as urban for codes 1 through 3, corresponding roughly to commuting access to metropolitan areas of 50,000 or greater residents, and as rural for codes 4 through 10, corresponding to commuting access to urban centers of less than 50,000 residents. All other data used in analyses and provided publicly are in their raw form.

### 3.4 Dataset de-identification

In order to deposit our data in a public repository, we applied commonly accepted approaches for the de-identification of biomedical and clinical trial data to our final analytic dataset [16,17]. Specifically, we relied on the “Safe Harbor” de-identification standard of the United States Health Insurance Portability and Accountability Act (HIPAA) Privacy Rule and associated guidance included in the Health Information Technology for Economic and Clinical Health (HITECH) Act [18]. The “Safe Harbor'' standard requires the removal of 18 identifiers related to an individual, their relatives, or their employer, comprising predominantly of personal identification numbers (e.g. social security number, medical record number), contact information (e.g. telephone numbers, IP addresses), date elements except year, geographic subdivisions smaller than a state, and any other unique identifying number, characteristic, information, or code that could be used either independently or in combination to re-identify an individual. Our detailed approach to filtering potentially identifying variables is outlined in the repository file *data_de-identifcation_steps_and_data_dictionary.xlsx.* In summary, we filtered raw data by removing all unique identification numbers, IP addresses, email addresses, dates, and condensed available geographic information into United States region instead of state to limit the potential for participant re-identification due to extremely small cell counts per unique combinations of age, race, ethnicity, medical specialty, and state.

## Limitations

Potential sampling limitations associated with this dataset include 1) the overall survey completion and data retention rate out of all valid email addresses reached (369/25,803, ∼1.4%), 2) possible selection bias associated with either or both opt-in to participate in the IQVIA ONEKEY database and consent to complete the survey described here, and 3) some observed differences in representation of racial, ethnic, and geographic groups in our data compared to other nationwide physician samples [19]. Such factors may limit complete representativeness of our data to all PCPs in the United States who may be anticipating or will be utilizing GRS in the future. Additionally, de-identification steps that resulted in less granular geographic areas may prohibit combining our data with other increasingly used and publicly available sociogeographic datasets such as The Neighborhood Atlas Area Deprivation Index [20]. Despite these constraints, this dataset comprises one of the first, largest, and broadest samples of United States PCP perceptions on the clinical decision-making, benefits, barriers, and utility of GRS to date.

## Ethics Statements

The protocol was approved by the Harvard Longwood Campus Institutional Review Board (Protocol #20-2098). All participants provided informed consent to participate in the study from which this data derive. Informed consent language associated with this project is included on the first page of each survey version (*qualtrics_survey_v1-4.pdf*).

## CRediT authorship contribution statement

**Charles A. Brunette:** Data curation, Formal analysis, Methodology, Software, Validation, Visualization, Writing – original draft, Writing – review & editing. **Elizabeth J. Harris:** Data curation, Investigation, Methodology, Project administration, Software, Validation, Writing – review & editing. **Ashley A. Antwi:** Project administration, Writing – review & editing. **Amy A. Lemke:** Methodology, Writing – review & editing. **Benjamin J. Kerman:** Conceptualization, Funding acquisition, Investigation, Methodology, Writing – review & editing. **Jason L. Vassy:** Conceptualization, Funding acquisition, Investigation, Methodology, Supervision, Writing – review & editing.

## Data Availability

Data from National PCP Survey on GRS (Original data) (Dataverse).
